# Long-term Implant Maintenance: A Systematic Review of Home and Professional Care Strategies in Supportive Implant Therapy

**DOI:** 10.1590/0103-6440202406178

**Published:** 2024-10-25

**Authors:** Tiago Guimarães Araújo, Cristiano Soares Moreira, Rodrigo Amigo Neme, Haipei Luan, Martinna Bertolini

**Affiliations:** 1 University of Pittsburgh, Department of Periodontics and Preventive Dentistry, Pittsburgh, Pennsylvania, United States of America.

**Keywords:** Leukocytes Mononuclear cells, Periodontitis, Pro-resolving lipid mediators, Trained innate immunity

## Abstract

The aim of this study is review the efficacy of different techniques of home care and professional care for long-term implant maintenance, when compared with their respective standard procedures (regular brushing or mechanical debridement with curette), in changing clinical parameters, such as bleeding on probing, probing depth, plaque score and gingival index, as reported in randomized clinical trials. Materials and Methods: A systematic literature search of randomized clinical trials was performed using the PubMed (MEDLINE), EMBASE and Cochrane library databases. A qualitative review was conducted to compare all the different techniques of home care and professional care for long-term implant maintenance. Results: Initial search involved a total of 816 articles, 233 via Pubmed (Medline), 306 via the Cochrane Library, and 483 via EMBASE, while an additional 16 articles were collected through manual screening. A total of 29 articles were assessed by full-text read for eligibility and a final count of 13 studies were included in systematic review. The results of the risk of bias assessment for the included RCTs according to the ‘RoB 2’. Results favored glycine powder air-polishing and ultrasonic devices over traditional mechanical debridement with curettes in improving clinical parameters. In at-home care, water flossers with chlorhexidine were able to reduce inflammation. Conclusions: Evidence points towards the use of glycine powder air-polishing and the use of ultrasonic devices for reduction of inflammation around implants, and for home care, many existing techniques seem to be able to control tissue inflammation, but the use of chlorhexidine in water-flossers seems to be a promising strategy.

## Resumo

Revisar a eficácia de diferentes técnicas de cuidados domiciliares e cuidados profissionais para a manutenção de implantes a longo prazo, em comparação com seus respectivos procedimentos padrão (escovação regular ou desbridamento mecânico com cureta), na alteração de parâmetros clínicos, como sangramento à sondagem, profundidade de sondagem, índice de placa e índice gengival, conforme relatado em ensaios clínicos randomizados. Materiais e Métodos: Foi realizada uma busca sistemática na literatura de ensaios clínicos randomizados utilizando as bases de dados PubMed (MEDLINE), EMBASE e Biblioteca Cochrane. Uma revisão qualitativa foi conduzida para comparar todas as diferentes técnicas de cuidados domiciliares e profissionais para a manutenção de implantes a longo prazo. Resultados: A busca inicial envolveu um total de 1038 artigos, 233 via PubMed (Medline), 306 via Biblioteca Cochrane e 483 via EMBASE, enquanto 16 artigos adicionais foram coletados por triagem manual. Um total de 29 artigos foi avaliado por leitura completa do texto para elegibilidade, e um número final de 13 estudos foi incluído na revisão sistemática. Os resultados da avaliação de risco de viés para os ECRs incluídos foram realizados de acordo com o ‘RoB 2’. Os resultados favoreceram o uso de polimento a ar com pó de glicina e dispositivos ultrassônicos em comparação com o desbridamento mecânico tradicional com curetas na melhora dos parâmetros clínicos. No cuidado domiciliar, irrigadores orais com clorexidina conseguiram reduzir a inflamação. Conclusões: As evidências apontam para o uso de polimento a ar com pó de glicina e o uso de dispositivos ultrassônicos para a redução da inflamação ao redor dos implantes, e, para o cuidado domiciliar, muitas técnicas existentes parecem ser capazes de controlar a inflamação dos tecidos, mas o uso de clorexidina em irrigadores orais parece ser uma estratégia promissora.

**Figure:**
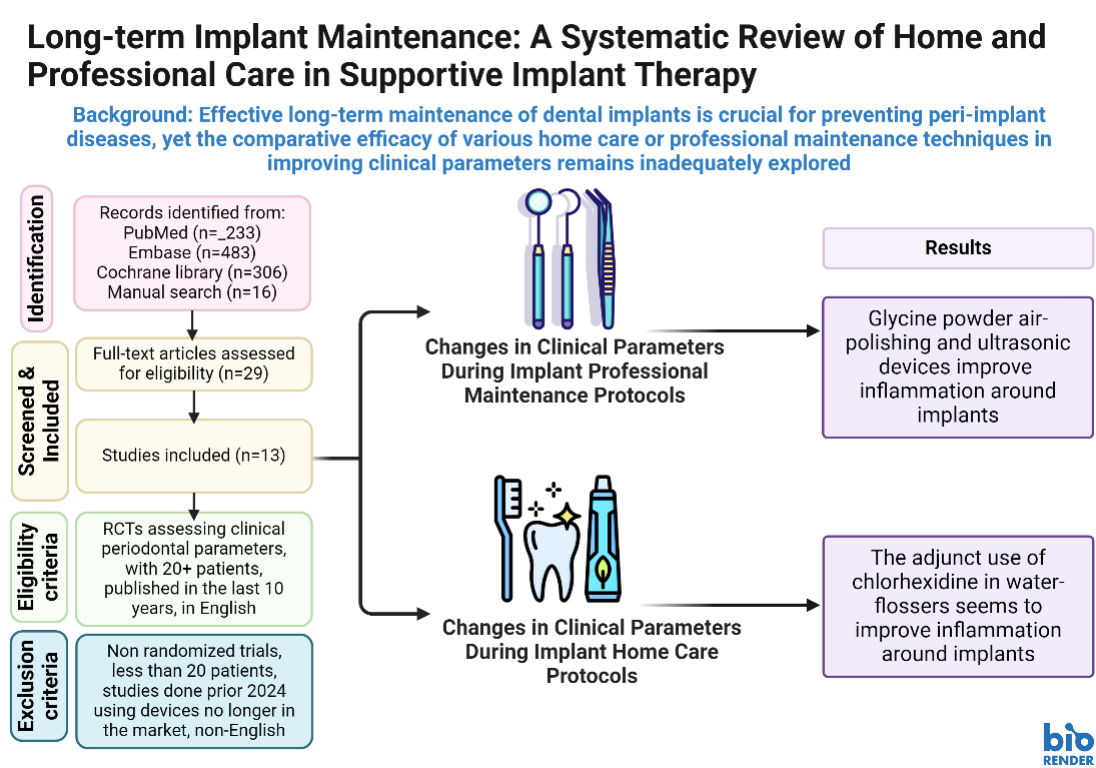

## Introduction

Implant rehabilitation treatments have become the preferred choice for replacing missing teeth in partially or fully edentulous patients over the last decades due to their high success rates. However, as the number of dental implant procedures increases, peri-implant diseases are becoming more common. The most common biological complication around dental implants is peri-implant mucositis, which can affect about 64.6% of all implant patients^1^. Peri-implant mucositis is an inflammatory condition that affects mucosal tissue around dental implants, and it shares similarities with gingivitis. If left untreated, it may progress to peri-implantitis, characterized by an inflammation in the peri-implant soft tissue and progressive loss of supporting bone, potentially leading to implant failure. 

There is strong evidence that there is an increased risk of developing peri-implantitis in patients who have a history of chronic periodontitis, poor plaque control skills, and no regular maintenance care after implant therapy^2^. Since mucositis precedes peri-implantitis, controlling plaque is crucial in preventing peri-implantitis^3^. A 5-year study revealed that those who attended maintenance therapy had an 18% incidence of peri-implantitis compared to 44% without professional care^4^. Moreover, when patients do not follow recommended maintenance therapy, they often need more peri-implantitis treatment over ten years (41%) compared to those who attended follow-up appointments (27%)^5^. The main goals of peri-implantitis treatment includes managing inflammation, preventing bone loss around the implant, and maintaining healthy peri-implant conditions. 

Implant-supported crowns are a standard treatment option for missing single teeth, with high clinical survival rates and long-term patient satisfaction. However, as the number of missing teeth increases, and the complexity of implant-supported prosthetic rehabilitation also rise, the final design can create hard-to-reach areas, making it challenging to support proper oral hygiene by patients with regular home using only toothbrushes. Therefore, it is necessary to use some other tools, such as dental floss, interdental brushes, and water flossers, to eliminate non-adherent bacteria and debris from the spaces under prosthesis with large pontic surfaces.

According to the latest position statement of the American College of Prosthodontists, effective maintenance strategies within supportive implant therapy for full arch rehabilitations involve a collaborative effort between patient and dentist. While patients are responsible for maintaining personal hygiene, dentists must ensure the prosthesis has hygienic contours and self-cleansable inter-implant pontic sections, following standard prosthodontic guidelines. Long-term success hinges on proper design, patient home care, and professional recall maintenance. While maintenance protocols should include regular evaluations of soft tissues for inflammation, assessment of plaque and calculus, baseline probing depths, and periodic radiographs; the professional maintenance involves educating patients on effective oral hygiene practices and using appropriate cleaning instruments. Finally, the removal of fixed prostheses is recommended only in specific cases of hygiene or mechanical complications^6^. 

The present study aims to review the efficacy of different techniques of home care and professional care for long-term implant maintenance, when compared with their respective standard procedures (manual brushing or mechanical debridement with curette), in changing clinical parameters, such as bleeding on probing, probing depth, plaque score and gingival index, as reported in randomized clinical trials. 

## Material and methods

### Study Registration

The review protocol was registered and given an identification number (CRD42024538084) in the PROSPERO International Prospective Register of Systematic Reviews, which is hosted by the National Institute for Health Research, University of York, Centre for Reviews and Dissemination.

### Patient, Intervention, Comparison, Outcome (PICO) Question

This systematic review utilized the Preferred Reporting Items Systematic Review and Meta-Analyses (PRISMA) statement and, as well as the population, intervention, comparison, outcomes (PICO) question, as follows: In patients with implants (population), what is the efficacy of home care and professional care for long-term implant maintenance (intervention) when compared with their respective standard procedures, such as manual brushing or professional mechanical debridement with curettes (comparison), in changing clinical parameters, such as bleeding on probing, probing depth, plaque score and gingival index as reported in randomized clinical trials (RCT) (study design)

- P - Patients with implants (aged over 18 years)
- I - Home care (regular toothbrush, floss, water flosser, interdental brush) or long-term professional care (air polishing, curettes, sonic instruments)
- C - The control groups used on the RCTs were considered controls; whenever possible, the control was considered regular home care with manual brush and the professional care was curettes.
- O - The main outcome was plaque index/gingival index and BoP.

### Information Sources and Screening Process

**Figure 1:**
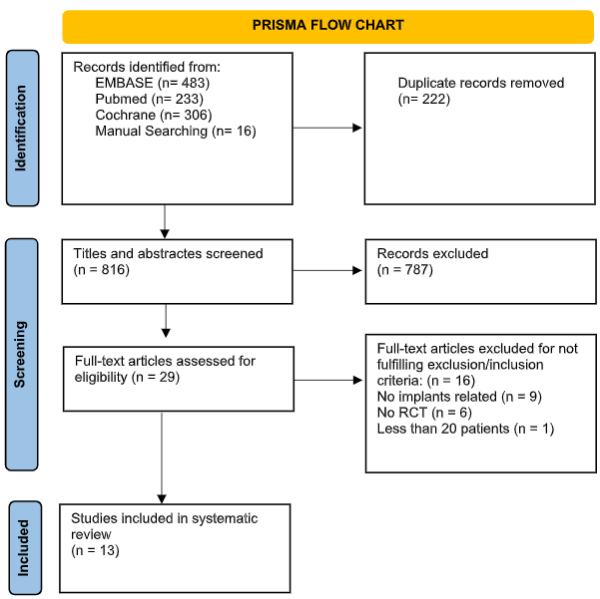
Study selection

Electronic and manual literature searches, conducted by two independent reviewers (C.M. and T.A.), covered studies until April 2024 across the National Library of Medicine (MEDLINE by PubMed), EMBASE and Cochrane. Additionally, a manual search of related journals was also performed. Finally, previous systematic reviews investigating implant maintenance protocols were screened for article identification (Figure 1 PRISMA flow chart).

### Eligibility Criteria

Studies were deemed eligible if they met the following criteria: RCT studies showing at least one of the following clinical parameters, plaque index, gingival index, bleeding on probing (BoP), probing depth, when comparing the effectiveness home care or professional care for long-term implant maintenance (intervention) with their respective standard procedures (manual brushing or mechanical debridement with curette), (comparison), 2) studies with a minimum of 20 patients included, 3) studies that evaluate clinical parameters, such as plaque index, gingival index, or bleeding on probing (BoP), probing depth, 4) studies published in the last 10 years, in order to reflect recent technological advancements, updated methodologies, or current standard practices, 5) studies published in the English language. 

### Exclusion Criteria

Studies were deemed eligible if they were case studies, reviews, nonrandomized trials, small sample size (less than 20 patients), studies done before 2014, in order to not include studies using devices already removed from the market (i.e. air abrasive devices using bicarbonate powder) studies published in non-English language.

### Data extraction

Two investigators (C.M. and T.A.) screened titles and abstracts and conducted full-text reading to exclude studies based on predetermined eligibility criteria. A data extraction form was used to confirm the eligibility of each study based on the criteria. The primary outcome was Plaque Index, while the secondary outcome was bleeding on probing (BoP). Two authors (C.M. and T.A.) independently extracted data, including patient characteristics, treatments, and clinical outcomes. In cases of missing clinical data, the trial authors were contacted. Disagreements between reviewers were resolved through discussion and consensus. If disagreements persisted, the judgment of a third reviewer (M.B.) was decisive.

### Quality and Risk of Bias Assessment

Two authors (H.L, T.A.) independently evaluated the included articles using all the checklist items of the respective parameters in RoB 2: A revised Cochrane risk-of-bias tool for randomized trials. RoB 2 is structured into a fixed set of domains of bias, focusing on different aspects of trial design, conduct, and reporting. Within each domain, a series of questions ('signaling questions') aim to elicit information about features of the trial that are relevant to risk of bias. A proposed judgement about the risk of bias arising from each domain is generated by an algorithm, based on answers to the signaling questions. Judgement can be 'Low' or 'High' risk of bias or can express 'Some concerns'.

## Results

### Study Selection

Initial search involved a total of 1038 articles, 233 via Pubmed (Medline), 306 via Cochrane Library, and 483 via EMBASE, while an additional 16 articles were collected through manual screening. However, 222 duplicate records were removed, leading to 787 titles and abstracts to be screened. Next, full-text articles were assessed for eligibility (n = 29), and 13 studies were included in the systematic review (Box 1). Removed articles were not included due to the following reasons: Full-text articles were excluded for not fulfilling exclusion/inclusion criteria (n = 16), No implants related (n = 9), No RCT (n = 6), Less than 20 patients (n = 1) (Figure 1, PRISM flow chart). 

### Quality Assessment

**Figure 2:**
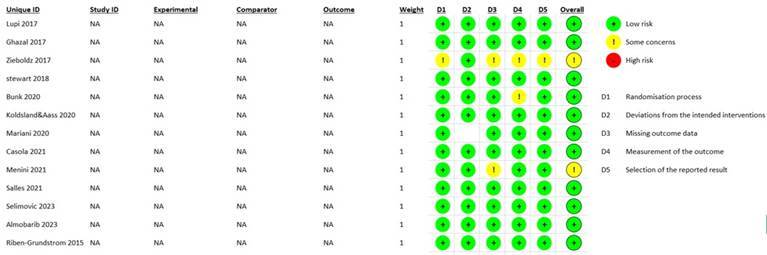
RoB2. A revised Cochrane risk-of-bias tool for randomized trials

The results of the risk of bias assessment for the included RCTs according to the RoB 2: A revised Cochrane risk-of-bias tool for randomized trials are summarized in figure 2. Two articles were considered as having ‘some concerns’ ^7^ ^,^ ^8^, all others were considered ‘low risk’^9^ ^,^ ^10^ ^,^ ^11^ ^,^ ^12^ ^,^ ^13^ ^,^ ^14^ ^,^ ^15^ ^,^ ^16^ ^,^ ^17^ ^,^ ^18^ ^,^ ^19^. Articles classified as "low risk of bias" in a Bias Assessment means that the study was conducted with minimal potential for bias, ensuring the credibility and reliability of its results. This classification indicates that the randomization process was appropriately conducted, deviations from intended interventions were minor, missing outcome data were properly handled, outcome measurements were unbiased, and all predefined outcomes were reported without selective reporting. Consequently, the findings of such a study are likely to accurately reflect the true effect of the interventions, providing greater confidence in the study's conclusions for researchers, clinicians, and policymakers.

### Changes in Clinical Parameters During Implant Professional Maintenance Protocols

*Non-Surgical Treatments with Glycine Powder Air-Polishing and Ultrasonic Devices*

Riben-Grundstrom et al.^16^ compared the effectiveness of glycine powder air-polishing and ultrasonic devices for the non-surgical treatment of peri-implant mucositis. The study concluded that both treatments were effective in reducing inflammation and the number of peri-implant pockets, provided patient compliance was maintained. Significant reductions were observed in all clinically measured variables, particularly in gingival bleeding and bleeding on probing (BOP), which are critical indicators of peri-implant disease. However, some sites remained diseased after 12 months, highlighting the challenge of achieving complete resolution of inflammation). Despite these positive outcomes, the study noted the need for longer follow-up periods to fully assess the long-term efficacy of these treatments.

### Effectiveness of Glycine Powder Air-Polishing in Comparison to Manual and Mechanical Debridement

Lupi et al. ^14^ compared traditional treatment with plastic curettes and chlorhexidine to treatment with glycine powder. The results indicated that glycine powder was more effective in maintaining peri-implant health and reducing peri-implant biofilm than manual instrumentation. Improvements in probing depth (PD), plaque index (PI), BOP, and bleeding score (BS) were more pronounced in the *Glycine Powder* ^14^. These findings align with previous study of Schwarz et al., demonstrating the safety and efficacy of glycine powder air-polishing for non-surgical treatment^20^.

### Comparison of Titanium Curettes and Glycine Powder Air-Polishing

Ghazal et al.^9^ compared titanium curettes to Glycine Powder Air-Polishing treatments, focusing on cytokine levels and peri-implant hygiene over 12 months. Both treatments resulted in low concentrations of IL-10, IL-12, and TNF, with no significant differences in their levels or trends over time. While both treatments were effective in reducing peri-implant inflammation, the study emphasized the importance of a longer follow-up period to better compare the debridement methods. The study's limitations included small sample size and the absence of data on implant connection types and surface characteristics, which could influence plaque retention and BOP levels ^9^.

### Combination Treatments: Curettes, Ultrasonic Devices, Glycine Powder Air-Polishing, and chlorhexidine Varnish

Zieboldz et al.^8^ evaluated the efficacy of various combinations of curettes, Cavitron, Glycine Powder Air-Polishing, and chlorhexidine (CHX) varnish. Group B (curettes, Glycine Powder Air-Polishing, and prophy brushes) showed a significant reduction in pocket probing depth, but no significant changes and bleeding on probing. The addition of CHX varnish did not provide significant short-term benefits. The study suggested that Glycine Powder Air-Polishing is safe for plaque removal, but further research is needed to explore the impact of implant location and patient age on treatment outcomes^8^. The study faced challenges with patient follow-up, which impacted the results' robustness^8^.

**Box 1:**
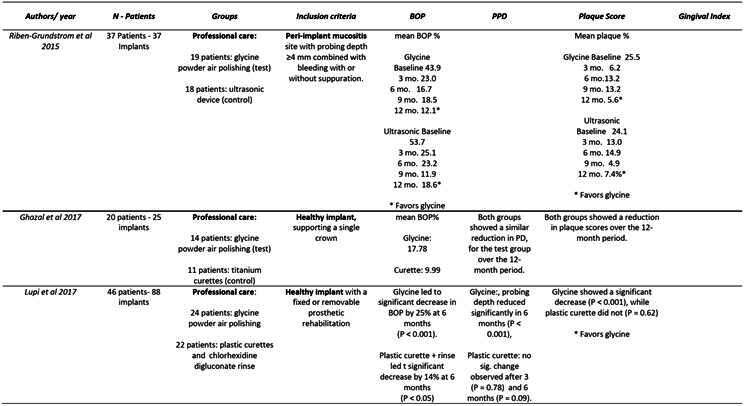
Data extraction

**Box 1:**
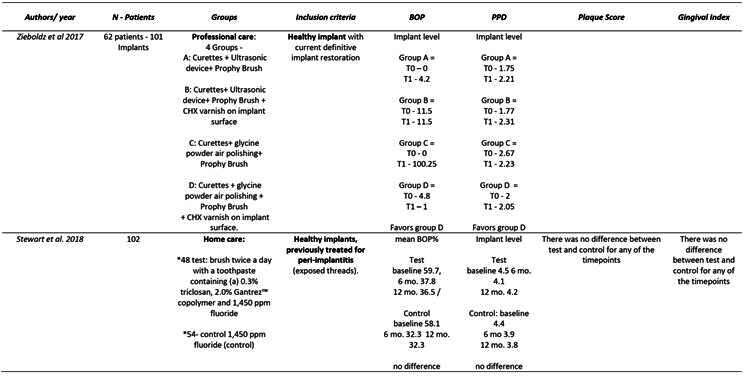
Continuation

**Box 1:**
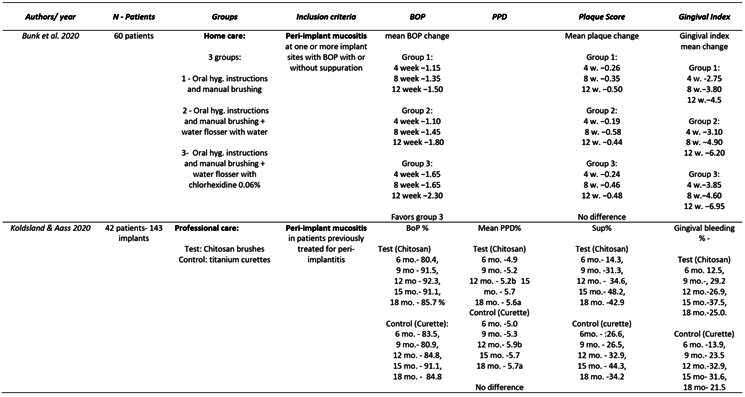
Continuation

**Box 1:**
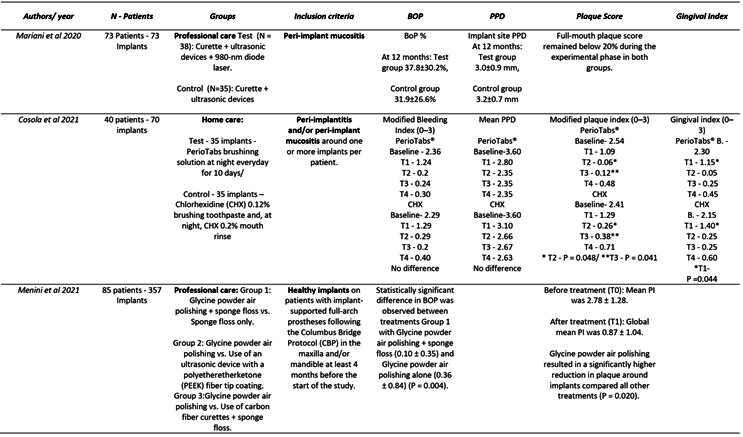
Continuation

**Box 1:**
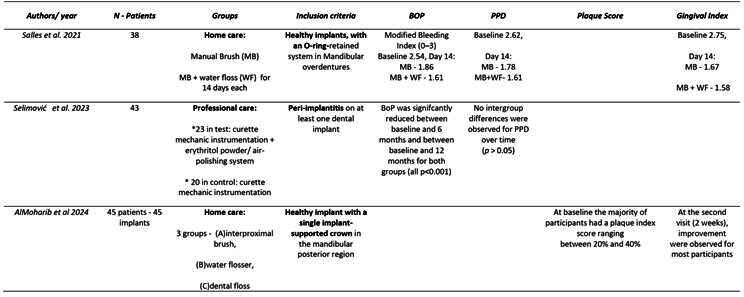
Continuation

### Efficacy of Titanium Curettes and Chitosan Brushes

Koldsland and Aass^13^ found no significant difference in the effectiveness of titanium curettes versus chitosan brushes in reducing peri-implant inflammation. The clinical status deteriorated throughout the observation period, highlighting the challenge of non-surgical treatment of peri-implantitis. The study compared its findings with previous research that showed better treatment outcomes, suggesting that the severity of bone loss and the inability to reach specific implant surfaces might have influenced the results. The study's limitations included the advanced stage of bone loss in subjects and the mechanical debridement's inefficiency in eliminating bacterial biofilm^13^.

### Diode Laser Adjunct to Mechanical Debridement

Mariani et al.^15^ examined the use of a diode laser (DL) as an adjunct to mechanical debridement (MD) in treating peri-implant mucositis. Both treatment modalities significantly reduced inflammation and probing depth, but the adjunct use of DL showed no statistically significant additional benefits. Kreisler et al.` study suggested that the presence of body fluids in inflamed pockets might influence DL's efficacy^21^. The main drawback was the lack of additional benefits from DL irradiation, and further research is needed to optimize its use in conjunction with mechanical debridement ^15^.

### Glycine Powder Air Polishing Versus Manual and Mechanical Treatments

Menini et al.^7^ conducted a split-mouth randomized controlled trial comparing glycine powder air polishing (GPAP) with manual curettes and mechanical treatments. GPAP was found to be an effective alternative, removing 74.5% of plaque deposits and significantly reducing plaque around implants. Manual curettes combined with sponge floss demonstrated the highest efficacy (84.8% plaque removal). The study highlighted GPAP's high patient comfort and its effectiveness in reducing plaque on both natural and prosthetic surfaces ^14^ ^,^ ^16^. The study recommended removing prostheses for optimal professional oral hygiene, although this might cause discomfort^7^.

**Figure 3:**
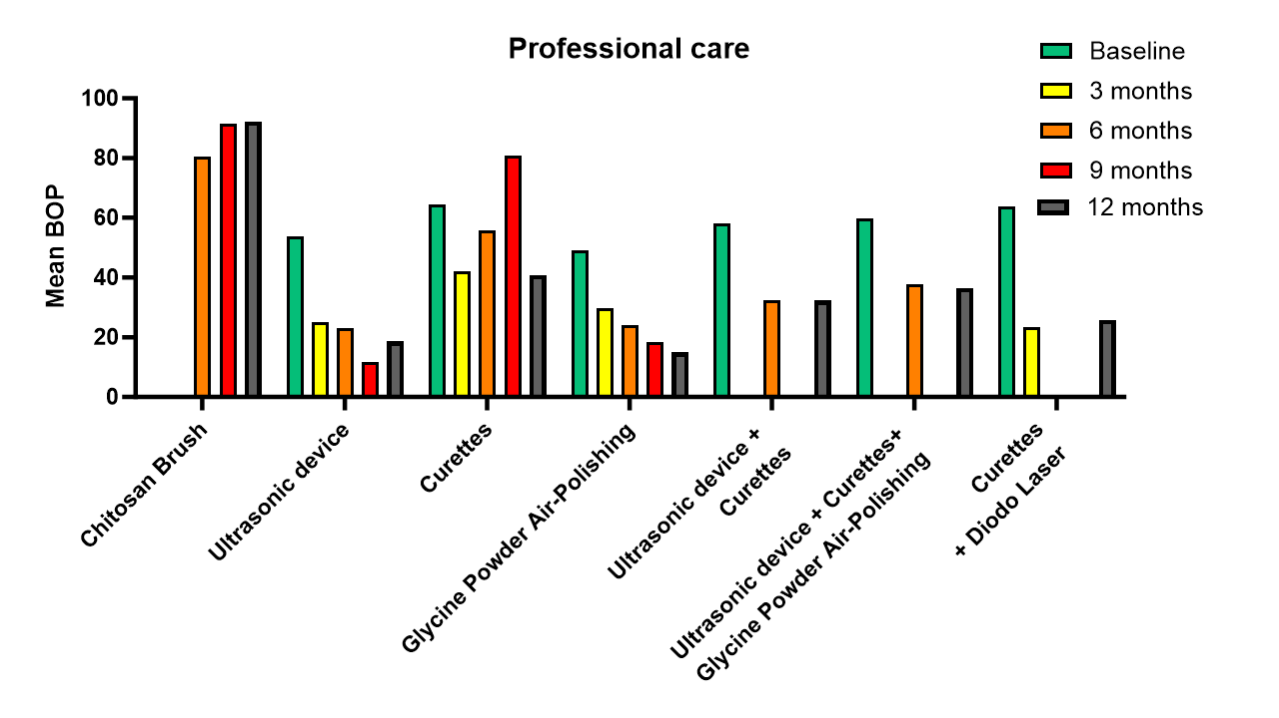
Mean Percentage of Bleeding on Probing (BOP) for Professional Implant Maintenance Methods. The graph highlights the reduction in mean BOP percentage from baseline to 12 months for each group. Notably, both Powder Air Polishing and ultrasonic devices demonstrate a sustained reduction in BOP, indicating their efficacy in managing peri-implant diseases

Overall, the mean percentage of bleeding on probing (BOP) was widely used surrogate marker for peri-implant inflammation, indicating the presence of inflammation in the tissues around dental implants, data from the cited studies were pulled together to create a graph (Figure 3), comparing the professional methods for implant maintenance and treatment, thus highlighting the reduction over time of mean BOP percentage, per group. Note how both, Powder Air Polishing and ultrasonic devices can keep a reduction from baseline to 12 months. Thus, BOP is valuable for early detection and monitoring of peri-implant diseases, and useful in assessing treatment efficacy, thus being used here.

###  Changes in Clinical Parameters During Implant Home Care Protocols

### Water flosser in Improving Peri-Implant Health

Salles et al.^17^ compared manual brushing with water flosser to manual brushing alone. Both methods significantly reduced clinical parameters compared to baseline, with the water flosser method showing slightly better improvements. The study supported previous findings on the benefits of oral irrigation in reducing clinical signs of gingivitis and periodontitis. The study noted no significant difference between manual brushing and water floss, indicating that both methods are effective in improving peri-implant health^17^.

### Water flossers carrying 0.06% chlorhexidine and Peri-Implant Mucositis

Bunk et al.^11^ explored the adjuvant use of an oral irrigator with 0.06% CHX in addition to mechanical biofilm removal. The study found that using an oral irrigator with CHX reduced the severity and presence of peri-implant mucositis more effectively than water irrigation alone. Despite side effects such as tooth staining and mucosal irritations, CHX's antimicrobial properties were beneficial. The interventions were highly compliance-dependent, and long-term studies are necessary to evaluate the sustained impact of oral irrigators ^11^.

### Impact of Triclosan and Gantrez Toothpaste on Peri-Implant Health

Stewart et al.^19^ investigated the effects of a toothpaste containing 0.3% Triclosan (antimicrobial) and 2% 2% PVM/MA copolymer (aiming to reduce biofilm adhesion and co-aggregation) on peri-implant health compared to conventional fluoride toothpaste. Over 24 months, the test group showed greater stability in clinical attachment and a reduction in bleeding on probing and probing depth around implants. The test group also experienced beneficial changes in subgingival biofilm composition, with a significant reduction in red complex pathogens. The study's limitation was the plaque index used, which may not be entirely suitable for implant surfaces due to staining issues with resin restorations and ceramic implants ^19^.

### PerioTabs

Cosola et al.^12^ investigated the efficacy of PerioTabs compared to chlorhexidine toothpaste and night rinse. Both groups showed significant improvements in clinical parameters, but the PerioTabs group had better outcomes for gingival index (GI) and full-mouth bleeding score (FMBS) after professional treatment^12^. The study confirmed that MFMD is effective for treating periodontal and peri-implant disorders, with PerioTabs providing superior results compared to chlorhexidine. The study noted that neither PerioTabs nor chlorhexidine should be used long-term, and recommended intermittent use of PerioTabs for maintenance ^12^.

### Interproximal brush, a water flosser, and dental floss

AlMoharib et al.^10^, in 2024, aimed to compare the effectiveness of an interproximal brush, a water flosser, and dental floss in removing plaque and reducing inflammation around implant-supported crowns. Plaque index scores, gingival index scores, and interleukin-6 (IL-6) levels were assessed at baseline and after two weeks. Results showed improvements in plaque control across all methods, with the water flosser demonstrating a slight but statistically insignificant reduction in IL-6 levels, while both the interdental brush and dental floss showed slight increases in IL-6 levels. Only the interproximal brush group showed a statistically significant difference in IL-6 levels (p=0.008). Overall, all three methods effectively improved plaque control and reduced gingival inflammation, with the water flosser indicating a potential advantage in reducing inflammation. The findings suggest further research is needed to evaluate the long-term efficacy and impact of these methods on implant survival.

**Figure 4:**
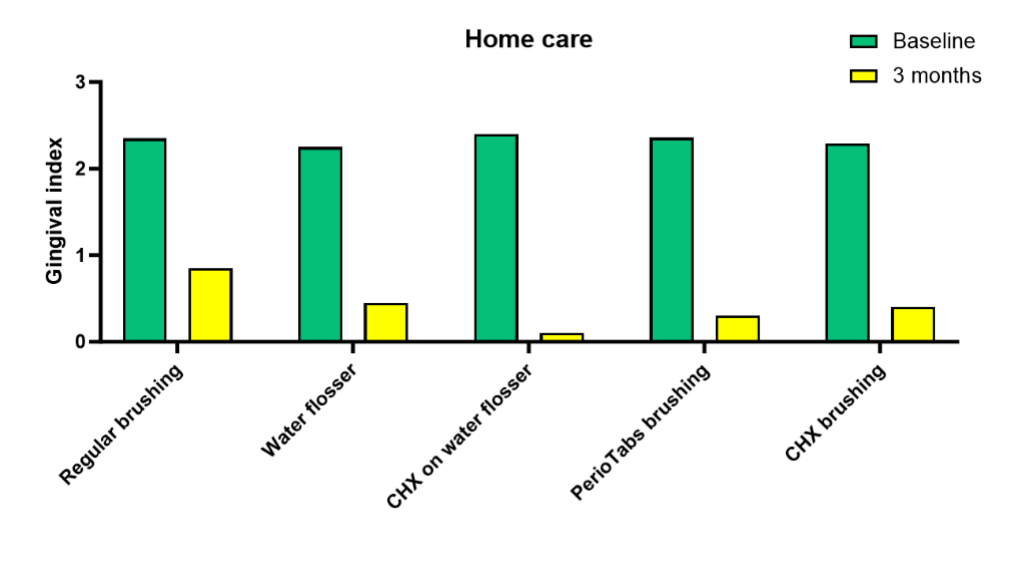
Gingival Index Scores for Home Care Methods in Peri-Implant Inflammation Assessment. The graph compares Gingival Index scores from baseline to 3 months for various home care methods discussed in the review. Notably, the incorporation of chlorhexidine in the water flosser appears to be a promising approach for maintaining gingival health around dental implants, as indicated by the observed improvements in the Gingival Index scores.

Importantly, the Gingival Index, traditionally used to assess gingival inflammation around natural teeth, was adapted and used by some of the authors cited above, as a surrogate marker for peri-implant inflammation. This standardized method allows for the quantification and monitoring of peri-implant tissue health, aiding in the evaluation of treatment effectiveness. Thus, it was used here for a more numerical and direct comparison from baseline to 3 months, regarding the home care methods discussed (Figure 4). Note how the use of chlorhexidine in the water flosser seems to be a promising method for maintenance of gingival health around implants.

## Discussion

The current existing protocols used for home care and professional care for long-term implant maintenance or treatment of peri-implant infections are often an overlooked topic when discussing implant management therapies. To the best of our knowledge, this is the first systematic review to interrogate what is the efficacy of different techniques of home care and professional care for long-term implant maintenance, when compared with their respective standard procedures (regular brushing or mechanical debridement with curette), in changing clinical parameters, such as bleeding on probing, probing depth, plaque score and gingival index, as reported in randomized clinical trials.

Overall, our results showed that using glycine powder air-polishing or an ultrasonic device effectively reduced bleeding on probing over time, although patient compliance in maintaining low plaque levels might have contributed to the positive outcomes. While complete cleaning of implant surfaces was not always achievable, glycine powder air-polishing showed superior results, but patient compliance and specific implant characteristics significantly influence long-term success. 

Specifically, Riben-Grundstron et al.^16^ has shown that non-surgical treatment with a glycine powder air-polishing or ultrasonic device is effective in reducing bleeding on probing continuously reduced throughout the study, however one possible reason for the positive outcome, besides the effect of therapy, could be related to the patient’s compliance in maintaining low plaque indices during the whole study period due to additional motivation to home care. Importantly, a in vitro model simulating different vertical bone angulations has shown that a complete cleaning of the implant surfaces was not possible in any of the defect models, highlighting that therapy results might vary depending on the access for the operator during air-polishing^22^. Moreover, when compared with traditional plastic curettes and chlorhexidine, glycine powder air-polishing seems more effective in the maintenance of peri-implant health ^14^. These results highlight that, even though access might be a limiting factor for glycine powder air-polishing, this could be even more limiting for conventional curettes. Still glycine powder air-polishing led to improvement of probing depth, plaque index, bleeding on probing and bleeding scores were noted at 3- and 6-months intervals. Authors have also highlighted that glycine powder air-polishing was not associated with any adverse effects, such as the formation of emphysema or site infection due to residues of glycine powder, and this observation is in line with the previous clinical trials ^16^ ^,^ ^20^ ^,^ ^23^ that confirmed the safety use of the device for the non-surgical treatment of peri-implant and periodontal soft tissue inflammation. Surprisingly, when cytokine levels were evaluated around peri-implant tissues, low concentrations of IL-10, IL-12, and TNF over the 12-month period, were noted for curettes and glycine powder air-polishing, suggesting that although clinical parameters favored air-polishing, the curettes were able to remove plaque and biofilm deposits enough to reach a threshold that led to an improvement in the host immune response (but with sub clinical results)^9^.

Thus, a combination of different mechanical techniques has also been proposed, but Zieboldz et al.^8^, 2017 showed that when ultrasonic devices or prophy brushes are used, the added effect of glycine powder air-polishing did not reveal any major improvements in the short-term compared to mechanical therapy, possibly indicating an overlap in efficacy for mechanical biofilm removal from implant surfaces among different mechanical methods of decontamination. Another combination of mechanical methods recently tested by Menini et al.^7^, 2021, used glycine powder air-abrasion and sponge floss, and although, as expected, glycine powder air polishing was found to be an effective decontamination method, authors also found that the use of manual curettes combined with sponge floss demonstrated the greatest efficacy in plaque deposit removal from implants/abutments, removing 84.8% of plaque deposits (although with no statistically significant difference when compared to other treatments) and glycine powder air polishing was the second most effective method for removing biofilm around implants/abutments (74.5%) and resulted in a statistically significant reduction of plaque around implants/abutments compared to the control treatments. Importantly, none of the professional hygiene treatments performed in the present study were able to completely remove plaque deposits from the implants or the prosthetic components. Consequently, the present authors recommend that prostheses are removed to ensure optimal cleansing, despite the discomfort this might cause. The possibility of achieving optimal professional oral hygiene thanks to prosthesis removal is one of the advantages of screw retention in implant prosthodontics; however, wear of the prosthodontic components (screws) should be carefully evaluated, and indications on the frequency of their replacement are missing in the literature.

More recent, alternating rotating subgingival brushes (Chitosan) have been developed and tested as an alternative mechanical biofilm removal method for biofilm and debris removal of implant surfaces subgingivally, without the need to flap elevation on non-surgical intervention. Although interesting, this method has been proven ineffective^13^, due to the inability for these instruments to reach the “valley areas” and “apically facing” thread surfaces of the implant threads, hampering the efficiency of the mechanical debridement of the implants, making it difficult to eliminate bacterial biofilm formation.

Regarding the use of lasers, the diode laser has been suggested as an adjunct to mechanical debridement ^15^, but no additional benefit had been found. Both treatment modalities resulted in a statistically significant reduction in inflammation and PD at peri-implant mucositis sites over a 12-month observation period. A complete disease resolution could not be achieved at all implant sites regardless of the instrumentation method applied. This also highlights the limitation of mechanical debridement in hard-to-reach areas, and corroborating this hypothesis, implants with supramucosal restoration margins showed greater improvement following the treatment of peri-implant mucositis compared with those with submucosal restoration margins. In the present study all test and control implants had an epimucosal positioning of the crown margin. Moreover, it is important to mention that, for a non-surgical procedure, diode laser irradiation might be influenced by the presence of several body fluids that are commonly associated with inflamed periodontal and peri-implant pockets, due to its affinity for hemoglobin and other pigments.

The application of topic antimicrobial agents, such as chlorhexidine varnish around implant sites have also been proposed. Zieboldz et al.^8^, 2017 showed that this had no significant benefit in reducing probing depth, and bleeding on probing around implants. In addition, the use of antimicrobial toothpaste (Triclosan 0.3% and 2% PVM/MA copolymer) also led to a better outcome when compared to regular toothpaste, helping clinical attachment stability and significantly reducing red-complex bacteria at 24 months ^19^. One limitation of this study was the plaque index used to evaluate plaque accumulation relying on a plaque disclosing solution, which although quite effective for natural teeth, are not as reliable for implant‐supported restorations made of ceramic, which normally accumulate less plaque and are harder to stain with disclosing solutions. Consequently, plaque in the implant surfaces was considerably low since the baseline assessment and could have therefore overestimated the results. Still on the use of local antimicrobials, Bunk et al., 2020 proposed the use of a water flosser loaded with 0.06% chlorhexidine for implant irrigation, and all participants could reduce signs of peri-implant mucositis, even the ones with regular water on water-flosser. Seventy percent of all study participants reached complete resolution of peri-implant mucositis, supporting the theory that complete resolution is possible when patient has compliance. Although its side effects, such as tooth staining, change of color of the mucosa and tongue surface, hypersensitivity, dryness of the mouth, mucosal irritations, or allergic reactions, Chlorhexidine is increasingly used in clinical practice due to its antimicrobial effects.

The full mouth disinfection technique, tested by Cosola et al.^12^, 2021, using PerioTabs® (Small effervescent tablets dissolved daily in lukewarm water forming a brushing solution with antimicrobial effect) showed that after 1 week of domestic intervention, the patients managed to reduce the amount of localized plaque, pain, and inflammation, while improving their overall oral health condition. Furthermore, all clinical parameters significantly improved in peri-implant mucositis sites, however, all the implants diagnosed with periimplantitis at baseline still presented with bleeding upon probing at the last follow-up visit. This highlights the need for further intervention, in addition to home care, for peri-implantitis treatment.

Finally, when looking into mechanical methods of home care for patients, Almoharib et al.^10^, 2024 compared the use of interproximal brushed, water flosser and regular dental floss for at home implant maintenance. The results show that all three interdental cleaning methods yielded notable improvements in plaque control, as demonstrated by reduced plaque index scores and decrease in bleeding on probing for most participants, although the water flosser demonstrated a noticeable reduction in IL-6 levels. Ng and Lim, suggested an advantage in reducing inflammation compared to the other groups, possibly due to the flushing action provided by the water flosser has a superior effect on the removal of bacterial plaque and the subsequent inflammatory process^24^. Importantly, a Cochrane review published in 2019 by Worthington & Clarkson^25^ found the effectiveness of water flossers, when compared with dental floss or interdental brushes, might be more effective in reducing bleeding.

Overall, many studies suggest combining different mechanical techniques to enhance outcomes, though no method completely removes plaque deposits. This highlights the importance of removing prostheses for optimal cleaning when too many hard to reach areas as present, such as in full arch prosthesis, in which disease implants are noted with bone loss. Adjunctive methods like the use of lasers, antimicrobial agents, and innovative tools have shown varying degrees of effectiveness, but patient compliance and specific implant characteristics significantly influence long-term success. One important note of the current studies, is that none of them took into consideration factors such as implant type (bone vs. tissue level), implant alloy material (titanium vs. zirconium based) and surface modifications of implants and prosthetic components (base metal, noble alloys or ceramics) all which could also have an impact on long term plaque accumulation and efficacy of decontamination methods, as plaque and bacteria adhesion rates differ depending on the type of restoration and material.

Another fact to be taken into consideration when thinking about the long term follow up results of these types of studies, is that most of home care procedures and techniques, regardless of the antimicrobial of mechanical method employed, are highly compliance-dependent and it can be assumed that the compliance level of the participants may naturally decrease after a certain time. Thus, constant motivation, and perhaps shorted intervals for office visit and professional maintenance procedures must be considered for patients who are unable to perform optimal home care maintenance on their implants. 

Finally, local factors, such as the presence of keratinized mucosal around implants, must also be considered when stablishing proper maintenance of dental implants, as the lack of keratinized mucosa is often associated with the severity of peri-implant mucositis. In addition, when thinking of peri-implantitis, the severity of bone loss has previously been described as a factor affecting the outcome of peri-implantitis treatment^13^. Further research is needed to establish more definitive treatment protocols for re-establishment and maintenance of peri-implant health.

It is important to consider that only studies published within the past 10 years were considered, to ensure the review encompasses the most up-to-date research, reflecting recent technological advancements, updated methodologies, and current standard practices. This timeframe excludes older studies where 'bicarbonate air-polishing' was still being tested, as glycine air-polishing dental devices started replacing bicarbonate around early 2010s, which was largely reflected in papers published in the last decade. However, this approach could have excluded other relevant studies that could have otherwise met the other inclusion criteria. A limitation of the current study if the fact that we restricted the inclusion to studies published in English, mostly due to translation resource limitations, and accessibility issues, but we consider that since the majority of high-impact journals in the relevant fields publish in English, and researchers from non-English speaking countries often publish their most critical work in English to reach a broader audience, not much was lost in other languages.

## Conclusion

This comprehensive review focused on the efficacy of different techniques of home care and professional care for long-term implant maintenance, when compared with their respective standard procedures (regular brushing or mechanical debridement with curette), in changing clinical parameters. There seems to be evidence pointing towards the use of glycine powder air-polishing and the use of ultrasonic devices for reduction of inflammation around implants, and for home care, many existing techniques seem to be able to control tissue inflammation, but the use of chlorhexidine in water-flossers seems to be a promising strategy. Long-term studies and more comprehensive assessments, including microbiological evaluations, are necessary to fully understand the benefits and limitations of these treatments. Further research is needed to establish more definitive treatment protocols for peri-implant health.

## References

[1] Heitz‐Mayfield LJA, Salvi GE, Mombelli A, Loup P, Heitz F, Kruger E (2018). Supportive peri‐implant therapy following anti‐infective surgical peri‐implantitis treatment: 5‐year survival and success. Clin Oral Implants Res.

[2] Schwarz F, Derks J, Monje A, Wang HL (2018). Peri-implantitis. J Periodontol.

[3] Jepsen S, Berglundh T, Genco R, Aass AM, Demirel K, Derks J (2015). Primary prevention of peri-implantitis: managing peri-implant mucositis. J Clin Periodontol.

[4] Costa FO, Takenaka-Martinez S, Cota LOM, Ferreira SD, Silva GLM, Costa JE (2012). Peri-implant disease in subjects with and without preventive maintenance: a 5-year follow-up. J Clin Periodontol.

[5] Roccuzzo M, Bonino F, Aglietta M, Dalmasso P (2012). Ten-year results of a three arms prospective cohort study on implants in periodontally compromised patients. Part 2: clinical results. Clin Oral Implants Res.

[6] Armitage GC, Xenoudi P (2016). Post-treatment supportive care for the natural dentition and dental implants. Periodontol.

[7] Menini M, Delucchi F, Bagnasco F, Pera F, Di Tullio N, Pesce P (2021). Efficacy of air-polishing devices without removal of implant-supported full-arch prostheses. Int J Oral Implantol Berl Ger.

[8] Ziebolz D, Klipp S, Schmalz G, Schmickler J, Rinke S, Kottmann T (2017). Comparison of different maintenance strategies within supportive implant therapy for prevention of peri-implant inflammation during the first year after implant restoration. Am J Dent.

[9] Al Ghazal L, O’Sullivan J, Claffey N, Polyzois I (2017). Comparison of two different techniques used for the maintenance of peri-implant soft tissue health: a pilot randomized clinical trial. Acta Odontol Scand.

[10] AlMoharib HS, AlAskar MH, Abuthera EA, Alshalhoub KA, BinRokan FK, AlQahtani NS (2024). Efficacy of Three Interdental Cleaning Methods for Peri-Implant Health Maintenance of Single Implant-Supported Crowns: A Randomised Clinical Trial. Oral Health Prev Dent.

[11] Bunk D, Eisenburger M, Häckl S, Eberhard J, Stiesch M, Grischke J (2020). The effect of adjuvant oral irrigation on self‐administered oral care in the management of peri‐implant mucositis: A randomized controlled clinical trial. Clin Oral Implants Res.

[12] Cosola S, Oldoini G, Giammarinaro E, Covani U, Genovesi A, Marconcini S (2022). The effectiveness of the information‐motivation model and domestic brushing with a hypochlorite‐based formula on peri‐implant mucositis: A randomized clinical study. Clin Exp Dent Res.

[13] Koldsland OC, Aass AM (2020). Supportive treatment following peri‐implantitis surgery: An RCT using titanium curettes or chitosan brushes. J Clin Periodontol.

[14] Lupi S, Granati M, Butera A, Collesano V, Rodriguez Y Baena R (2017). Air‐abrasive debridement with glycine powder versus manual debridement and chlorhexidine administration for the maintenance of peri‐implant health status: a six‐month randomized clinical trial. Int J Dent Hyg.

[15] Mariani GM, Ercoli E, Guzzi N, Bongiovanni L, Bianco L, Romano F (2020). One-year clinical outcomes following non-surgical treatment of peri-implant mucositis with adjunctive diode laser application. Minerva Stomatol.

[16] Riben-Grundstrom C, Norderyd O, André U, Renvert S (2015). Treatment of peri-implant mucositis using a glycine powder air-polishing or ultrasonic device: a randomized clinical trial. J Clin Periodontol.

[17] Salles MM, De Cássia Oliveira V, Macedo AP, Silva-Lovato CH, De Freitas De Oliveira Paranhos H (2021). Effectiveness of Brushing Associated With Oral Irrigation in Maintenance of Peri-Implant Tissues and Overdentures: Clinical Parameters and Patient Satisfaction. J Oral Implantol.

[18] Selimović A, Bunæs DF, Lie SA, Lobekk MAa, Leknes KN (2023). Non-surgical treatment of peri-implantitis with and without erythritol air-polishing a 12-month randomized controlled trial. BMC Oral Health.

[19] Stewart B, Shibli JA, Araujo M, Figueiredo LC, Panagakos F, Matarazzo F (2018). Effects of a toothpaste containing 0.3% triclosan in the maintenance phase of peri-implantitis treatment: 2-Year randomized clinical trial. Clin Oral Implants Res.

[20] Schwarz F, Becker K, Renvert S (2015). Efficacy of air polishing for the non-surgical treatment of peri-implant diseases: a systematic review. J Clin Periodontol.

[21] Kreisler M, Al Haj H, d’Hoedt B (2005). Clinical efficacy of semiconductor laser application as an adjunct to conventional scaling and root planing. Lasers Surg Med.

[22] Sahrmann P, Ronay V, Schmidlin PR, Attin T, Paqué F (2014). Three-dimensional defect evaluation of air polishing on extracted human roots. J Periodontol.

[23] Renvert S, Lindahl C, Roos Jansåker AM, Persson GR (2011). Treatment of peri-implantitis using an Er:YAG laser or an air-abrasive device: a randomized clinical trial. J Clin Periodontol.

[24] Ng E, Lim LP (2019). An Overview of Different Interdental Cleaning Aids and Their Effectiveness. Dent J.

[25] Worthington HV, MacDonald L, Poklepovic Pericic T, Sambunjak D, Johnson TM, Imai P, Clarkson J (2019). Home use of interdental cleaning devices, in addition to toothbrushing, for preventing and controlling periodontal diseases and dental caries. Cochrane Database Syst Rev.

